# Association Between Prevention Focus and Sedentary Behavior in Older Adults: Cross-Sectional Study

**DOI:** 10.2196/63280

**Published:** 2025-05-01

**Authors:** Jethro Raphael Suarez, Amber Blount, Kworweinski Lafontant, Joon-Hyuk Park, Rui Xie, Nichole Lighthall, Ladda Thiamwong

**Affiliations:** 1 Department of Mechanical and Aerospace Engineering, University of Central Florida, Orlando, FL, United States; 2 College of Nursing, University of Central Florida, Orlando, FL, United States; 3 Department of Counselor Education and School Psychology, University of Central Florida, Orlando, FL, United States; 4 Institute of Exercise Physiology and Rehabilitation Science, University of Central Florida, Orlando, FL, United States; 5 Department of Robotics and Mechatronics Engineering, Daegu Gyeongbuk Institute of Science and Technology, 333 Techno Jungang-daero, Hyeonpung-eup, Dalseong-gun, Daegu, 42988, Republic of Korea, 82 53-785-6233; 6 Disability, Aging, and Technology Cluster, University of Central Florida, Orlando, FL, United States; 7 Department of Statistics and Data Science, University of Central Florida, Orlando, FL, United States; 8 Department of Psychology, University of Central Florida, Orlando, FL, United States

**Keywords:** accelerometry, regulatory focus theory, actigraph, motivation, physical activity

## Abstract

**Background:**

Older adults engage in increased amounts of sedentary behavior (SB), which can result in a significant decline in muscle function and overall health. An understanding of the motivational driving factors that lead older adults to engage in SB can help to create effective intervention programs.

**Objective:**

This study aimed to determine the association between prevention and promotion focus with SB in older adults, as well as compare these associations with two factors (ie, age and BMI) that are commonly known to have an association with SB among older adults.

**Methods:**

A cross-sectional analysis was conducted among 93 community-dwelling older adults with a mean age of 74.98 (SD 6.68) years. Prevention and promotion focus were both assessed using the Regulatory Focus Questionnaire. Correlation analysis was performed to determine the associations between prevention focus, promotion focus, age, and BMI with SB. Anderson-Darling tests confirmed nonnormal data distributions for all factors (except age); therefore, Spearman rank correlation was used to determine correlations between factors. Comparative analysis of significant correlations was performed using Fisher Z transformation.

**Results:**

Prevention focus had the greatest statistically significant correlation with SB (ρ=0.296; *P*=.004), followed by BMI (ρ=0.204; *P*=.049). Both age (ρ=0.116; *P*=.27) and promotion focus (ρ=0.002; *P*=.99) had statistically insignificant correlations with SB, indicating no associations. The correlation between prevention focus and SB did not significantly differ from the correlation between BMI and SB (*P*=.51).

**Conclusions:**

Prevention focus was found to have a weak, but significant positive association with SB in older adults. Although age and BMI have been found to have an association with SB in previous literature, age was not associated with SB in this study, while BMI had a significant but relatively weaker association with SB than that with prevention focus. However, the association found between BMI and SB did not statistically differ from the association found between prevention focus and SB. These findings suggest that older adults could be driven to engage in increased amounts of SB due to having a dominant prevention focus, which revolves around thoughts of safety and avoiding negative consequences. The recognition of this association has the potential to aid in developing intervention programs that could promote shifting from prevention to promotion focus, thereby reducing SB in older adults.

## Introduction

Sedentary behavior (SB) is most prevalent among older adults and negatively affects their physical health by increasing their risk for diseases, as well as overall mortality [1]. SB can be defined as any waking behavior that results in an energy expenditure of less than 1.5 metabolic equivalents while in a sitting or reclining position [2-4]. Various intervention programs that aim to reduce SB in older adults have been developed and focus primarily on changing psychological and behavioral factors, rather than environmental or physical factors [5]. These interventions include self-regulatory strategies, which include goal setting and behavioral feedback with participants [6-8]. Self-regulation is described as the process of aligning an individual’s behavior and decision-making with their goals [9]. Regulatory Focus Theory builds on the idea of self-regulation by suggesting that individuals have two distinct motivational orientations that influence their decision-making and behavior: promotion and prevention focus [9-11]. Individuals with a prevention focus strive for feelings of safety and responsibility, and seek to minimize the negative outcomes or consequences in their lives (i.e., avoid losses) [10,11]. Conversely, individuals with a promotion focus strive for growth and advancement, seek opportunities for success, aim to maximize potential gains, and strive to obtain positive outcomes in life [10,11].

Regulatory focus theory has been found to relate to older adults, as evidenced by a positive relationship between negative self-perceptions on aging (ie, a pessimistic or unfavorable view toward aging) and trait prevention focus [12]. Negative self-perceptions of aging in older adults have also been found to have a negative relationship with self-reported physical activity (PA) and performance in daily activities [13-15]. This is likely due to a belief that PA and overall movement are not beneficial in the long term and could increase their risk of falling. Although regulatory focus theory has not yet been directly linked to SB in older adults, it has the potential to be related to SB, due to the previously found relationships with negative self-perceptions of aging. Establishing a link between regulatory focus theory and SB in older adults could potentially provide a better understanding of the motivational driving factors that lead older adults to engage more in SB.

Therefore, the aim of this study was to determine the association between prevention and promotion focus with SB in older adults and to compare these associations with two factors that are commonly known to be associated with SB among older adults (ie, age and BMI. We hypothesized that SB would have a significant positive association with age, BMI, and prevention focus, but a nonsignificant association with promotion focus.

## Methods

### Study Design

This cross-sectional investigation was part of a larger study that is federally funded by the National Institute on Minority Health and Health Disparities (R01MD018025) and has been pre-registered on ClinicalTrials.gov (NCT05778604). The protocol for the study has been previously published elsewhere [16]. The study was conducted in the Greater Orlando, metropolitan area, FL and recruitment was accomplished through word-of-mouth, flyer distribution, and partnership with local communities. A total of 141 community-dwelling older adults were recruited. Participants were included in this study if they were at least 60 years of age, could stand on their own, had low-income status based on the 2019 poverty thresholds relative to family size [17], had fully completed the Regulatory Focus Questionnaire (RFQ), and had at least 4 days of valid recorded ActiGraph data (each day≥10 hours of wear time). Participants who were unable to perform PA, receiving treatment from a rehabilitation facility, or hospitalized more than 3 times in the past 12 months were excluded. After screening for inclusion criteria, 93 participants were included and analyzed in this study.

### Data Sources

#### Demographic Measurements

Demographic characteristics such as age and sex were collected using a self-reported survey. Height and weight were assessed without shoes using a digital physician scale with a built-in stadiometer (Health-O-Meter, Model 402KL, McCook). BMI was calculated using height and weight as kg/m^2^.

#### Regulatory Focus Questionnaire

Participants were asked to complete the RFQ, an 11-item questionnaire that helps determine whether an individual’s motivational orientation is primarily prevention- or promotion-focused [18,19]. The RFQ produces independent prevention and promotion focus scores. Six questions contribute to the calculation of the promotion focus score, while five contribute to the prevention focus score. Seven questions are reverse scored (three for promotion and four for prevention). The RFQ uses a 5-point Likert scale based on how frequently specific events occur or have occurred in an individual’s life, ranging from 1 (“never or seldom”/ “never true”/ “certainly false”) to 5 (“very often”/ “very often true”/ “certainly true”). An example of a promotion question on the RFQ is as follows: “Compared to most people, are you typically unable to get what you want out of life?” The type of motivational focus most dominantly exhibited by an individual is based on the higher of the two scores (ie, prevention or promotion). For the prevention domain, a higher score indicates a greater individual focus on prevention (eg, individuals focus on avoiding negative outcomes and ensuring safety when making decisions). Similarly for the promotion domain, a higher score reflects a greater findividual focus on promotion (eg, maximizing potential gains and seeking growth and advancement). The RFQ has internal reliability coefficients of *α*=0.73 for the promotion scale and *α*=0.80 for the prevention scale [18].

#### Sedentary Behavior

To objectively measure SB, an ActiGraph GT9X Link wireless activity monitor (ActiGraph LLC) was used. The GT9X Link is a small and lightweight device (3.5×3.5×1 cm; 14g) that contains a triaxial accelerometer, has a dynamic range of ±8 gravitational units (g), and can be worn on either the hip or wrist. This device was initialized to record at a sampling rate of 30Hz. Participants were instructed to wear the device on their nondominant wrist for 7 consecutive days and to remove it only during showering or bathing. Accelerometer data were extracted from the GT9X Link using the ActiLife (version 6.13.5; ActiGraph LLC), R statistical software (version 4.3.1; R Foundation for Statistical Computing), and existing accelerometer data analysis code that use the *GGIR* R-package (version 3.1‐0) [20-23].

Total recorded SB (in minutes) was determined using the Euclidean Norm Minus One acceleration scalar metric in milli-gravitational units (mg) and classified as <30 mg [20]. Total SB was calculated for this study by summing the amount of SB (in minutes) for all valid days for each participant. For this investigation, participants were included if they had a minimum of 4 recorded days, with a minimum of 10 hours (600 minutes) each day, and a maximum of 7 recorded days. These requirements were consistent with previous PA research using the ActiGraph GT9X Link [24]. Data filtering was performed using the MATLAB multiparadigm program.

### Statistical Analyses

Minitab (version 21.4.3; Minitab LLC) and R software (version 4.3.1; R Foundation for Statistical Computing) were used to perform statistical analyses for this study. Each factor was checked for normality before analysis using descriptive statistics and Anderson-Darling normality tests through Minitab. Spearman rank correlation coefficients (ρ) were used due to the nonnormal distribution of each variable. R software was used to calculate correlation coefficients, compare statistically significant correlation values using Fisher Z transformation, and perform power analysis using the *pwr* package (version 1.3‐0) [25]. The threshold for statistical significance was set at *P*<.05.

### Ethical Considerations

This study was approved by the Institutional Review Board of the University of Central Florida (STUDY00003206), preregistered on ClinicalTrials.gov (NCT05778604), and carried out in accordance with the Declaration of Helsinki. The protocol has also been previously published elsewhere [16]. All participants gave written informed consent prior to participation. The data for this study were stored and managed using Research Electronic Data Capture (REDCap), a secure, web-based application that is designed to store data for research studies [26,27], and all participant data were deidentified. Participants were compensated US$50 in the form of a gift card upon completion of the study.

## Results

After screening for inclusion criteria, a total of 93 participants were included in this analysis, with the majority being female participants (83/93, 89%). Further self-reported demographic information regarding participants (eg, education level and general health) is shown in Table 1. Sixty-seven percent (62/93) of participants had a dominant prevention focus (as indicated by a greater prevention score), while 33% (31/93) of participants had a dominant promotion focus. Descriptive statistics for each factor are presented in Table 2.

**Table 1.** Participant demographics of community-dwelling older adults.

Variables	Participants (N=93)
Age (years), mean (SD), range	75.44 (6.71), 61.76‐89.46
Sex, n (%)	
Male	10 (11)
Female	83 (89)
Education, n (%)	
Lower than high school	14 (15)
High school	52 (56)
College or above	27 (29)
General health, n (%)	
Poor	1 (1)
Fair	15 (17)
Good	53 (57)
Very good	21 (23)
Excellent	2 (2)
Living status, n (%)	
Alone	65 (70)
With partner/spouse	13 (14)
With family/ friend	13 (14)
Other	2 (2)
Financial status, n (%)	
Much less than adequate	7 (8)
Less than adequate	20 (22)
Just enough	54 (58)
More than enough	11 (11)
Much more than enough	1 (1)

**Table 2.** Descriptive statistics for age, BMI, RFQ^a^ scores (prevention and promotion scales), and total SB^b^ (prevention and promotion groups) of community-dwelling older adults^c^.

Variables	Median (IQR)	Min	Max	*P* value^d^
Age (years)	74 (10)	61	89	.24
BMI (kg/m^2^)	29.77 (8.22)	20.95	47.03	.02
RFQ^a^ Prevention Focus Score	3.80 (1.20)	1.40	5.00	.01
RFQ Promotion Focus Score	3.33 (0.83)	1.67	4.67	.005
Total SB^b^ of Prevention Focus Group (min)	4989 (949)	2605	7713	.25
Total SB of Promotion Focus Group (min)	4851 (981)	2527	7660	.03

a RFQ: Regulatory Focus Questionnaire.

b SB: sedentary behavior.

c Due to having a normal distribution, the mean and standard deviation for the age factor (as opposed to the median and IQR) are presented elsewhere.

d *P* value represents the results from the Anderson-Darling normality tests (*P*<.05 indicates nonnormal distribution).

Correlational data is presented in Table 3 . The prevention focus score from the RFQ had the highest statistically significant correlation with total SB among the considered factors (ρ=0.296; *P*=.004). BMI had the second highest statistically significant correlation with total SB (ρ=0.204, *P*=.049). It was found through Fisher Z transformation that the correlation between prevention focus and SB was not significantly higher than that between BMI and SB (*P*=.51). Overall, the significant associations were found to be relatively weak. The association between RFQ promotion focus score and total SB was nonsignificant and near zero (ρ=0.002, *P*=.99). Age also had a weak, nonsignificant association with total SB (ρ=0.116, *P*=.27). Power analyses indicated that only the association between prevention focus and SB achieved adequate power of at least 80% (82.86%), exceeding the required sample size needed for achieving the Spearman coefficient of 0.296 (N=84). All other associations required a sample size that exceeded the total study sample size of 93 participants to achieve adequate power and therefore had relatively lower and inadequate power (<50%).

**Table 3.** Associations between age, BMI, RFQ^a^ prevention score, and RFQ promotion score with SB^b^.

Variables	Spearman rank correlation (ρ)	*P* value
Age	0.116	.27
BMI	0.204	.049
RFQ^a^ prevention focus score	0.296	.004
RFQ promotion focus score	0.002	.99

a RFQ: Regulatory Focus Questionnaire.

b SB: sedentary behavior.

## Discussion

### Principal Findings

The purpose of this study was to determine the association between prevention and promotion focus with SB in older adults, as well as to compare those associations with two factors that are commonly known to have an association with SB among older adults(ie, age and BMI). Analysis showed that SB has a significant positive association with BMI and prevention focus. This finding supports our initial hypothesis. Consistent with the hypothesis, promotion focus was not significantly associated with SB. Contrary to expectations, we did not observe an association between age and SB. BMI was associated with SB, but this association with SB was relatively weaker than that of prevention focus. Overall, the associations were weaker than expected. Comparative analysis of the significant correlation values using Fisher Z transformation showed that the correlation between prevention focus and SB was not significantly higher than the correlation between BMI and SB.

A key finding of this study is the observation of a weak, but positive association between a prevention-focused regulatory style and SB, indicating that individuals with a primarily prevention-focused mindset may engage in increased amounts of SB. This result may be attributed to the cautious, safe, and loss-avoidant mindset associated with the prevention focus orientation. This result aligns with previous work, which found that safety concerns of older adults are a barrier to reducing their sedentary time, whether in their own homes or in their neighborhoods [28,29]. Individuals that exhibit such personality traits may be more likely to avoid engaging in PA and overall movement due to the possibility of injury. An injury may result in losses, specifically the possible expenses associated with recovery, as well as the loss of time needed for physical recovery and possible loss of independence. Individuals with such mindsets may view PA or increased movement as dangerous or unsafe, thus leading to avoidance. Environmental factors such as a neighborhood safety can also contribute to this mindset, further preventing PA and movement outside of one’s home if an individual perceives that their safety in their neighborhood could be at risk.

The promotion focus scores and total SB were weakly associated, which may have been attributed to the possibility that decreased SB, as well as the benefits associated with it, are not considered as growth or advancement to older adults. Furthermore, older adults’ decision to engage in SB may be more influenced by a prevention focus because the desires associated with it (eg, desires for safety, avoidance of negative consequences, and avoidance of losses) are perceived as more of a “need” rather than a desire. In contrast, the desires associated with a promotion motivation orientation (eg, desire to achieve growth, advancement, and maximize potential gains), which could be considered as less of a “need.”

It was expected that BMI would have a significant positive association with SB, due to the directly proportional association between BMI and weight. Increased time in SB can potentially lead to weight gain and has been positively related to the risk of obesity and diabetes [30-33]. Furthermore, studies have shown that SB is positively associated with BMI [34,35]. Interestingly, despite the difference being statistically insignificant, the prevention focus scores from the RFQ had a relatively greater correlation coefficient value than BMI when compared to total SB. For years, BMI has been used as an index of obesity to classify individuals based on their body weight relative to height. BMI continues to be used in medical practice and research due to being an affordable and readily obtained metric [36]. However, BMI has faced numerous backlashes, primarily due to its inability to differentiate between body lean mass and body fat mass [36-39]. For example, an individual with a greater amount of body lean mass could have the same BMI as an individual with a greater amount of body fat mass. This variability present in BMI could explain the relatively lower correlation value when relating to SB. Furthermore, the motivation orientation of an individual, specifically an older adult can influence their decision-making and behavior, which could drive them to engage in SB, therefore directly resulting in changes to their BMI.

Various studies have shown a positive correlation between age and SB in older adults [40,41]. However, a nonsignificant association between the two variables was observed in this study. Previous work has found that a multitude of factors contribute to the SB levels of older adults beyond age, such as accessibility to transportation, living environment, and weather [42]. The sites from which participants in this study were recruited from, varied in terms of social life. Some sites held weekly social activities (eg, exercise classes and bingo nights), while others did not hold any social events. These differences could adversely affect the decision of older adults to engage in SB and could explain the weak and nonsignificant association between age and total SB in this study.

### Strengths, Limitations, and Future Directions

Overall, despite the weak associations, the findings of this study show that the characteristics associated with a prevention focus may be a factor that leads older adults to engage in SB. The results from this study support the idea that older adults might be less inclined to engage in SB if they are given suggestions on how to maintain safety during PA or reassured that it is acceptable to make mistakes. This idea can be used to create new or improve existing intervention programs aimed toward improving the PA levels of older adults, which can ultimately result in a reduction in their fall risk. Spearman rank correlation was chosen for the analysis of this study due to its ability to adequately manage both normally and nonnormally distributed data. Although an appropriate test was used to perform correlation analysis, certain limitations exist within this study. First, a power analysis showed that only the association between the prevention focus score from the RFQ and total SB produced adequate power (82.86%). Although this value indicates reasonable sensitivity for detecting the observed association, all other associations produced a power significantly below this value (<50%).

Second, this study did not identify the causal relationship between the considered factors and only focused on pairwise correlations, as opposed to considering confounding effects. For example, there was a significantly greater number of females included in the sample than male participants (83/93). It has been previously found that female participants tend to be more prevention-focused than male participants [43], which explains the larger number of prevention-focused individuals (62/93). The exploration of confounding effects could provide a deeper understanding of the associations presented in this study. Future work should involve increasing the sample size to include more promotion-focused individuals and raising the power of the correlation tests to adequately determine the associations between the factors, as well as using techniques such as regression to incorporate confounders and conduct formal causal analysis.

### Conclusions

Although many environmental factors drive older adults to engage in SB, psychological factors also play a role in influencing this behavior. Prevention focus, as determined by the RFQ was found to have a weak but significant positive association with SB in older adults. The association between BMI and SB was relatively weaker in comparison, but no significant differences were found between the two associations. These findings suggest that older adults may be driven to engage in increased amounts of SB due to having a dominant prevention focus, which revolves around thoughts of safety and the avoidance of negative consequences. Identifying an older adult’s motivational orientation can provide useful insight into understanding their decisions to engage in SB and further identify what strategic approach would be most effective when setting goals and implementing safeguards to decrease SB.

## Abbreviations

PA: physical activity
REDCap: Research Electronic Data Capture
RFQ: Regulatory Focus Questionnaire
SB: sedentary behavior
